# Supplementary material for: CRISPR analysis suggests that small circular single-stranded DNA smacoviruses infect Archaea instead of humans
Source: Nat Commun. 2019 Jan 17;10:294. doi: 10.1038/s41467-018-08167-w (PMC6336856; doi:10.1038/s41467-018-08167-w)
Supplement: Supplementary file 1 — Supplementary Information [file 41467_2018_8167_MOESM1_ESM.pdf]

Supplementary Information

"CRISPR analysis suggests that small circular single-stranded DNA smacoviruses infect *Archaea* instead of humans"

Díez-Villaseñor et al.

Supplementary Table 1. Representatives of best matches in smacoviruses to spacers in *Ca.* M. intestinalis.

Spacer	Target genome	Gene[a]	Identity	E-value	Upstream[b]	Downstream[c]	Alignment[d]
sp1	KP264968	-	88.9	$4.7\cdot10^{-10}$	AACCCTTCGA	CGCTCGAGAG	36 tactatatctctttggattcgttcagctcgagccgc 1<br>307 tactatatctcttgggatgcgttcagctagaaccgc 342
sp6	KP233175	-	100	$2.1\cdot10^{-17}$	AGGCACACCT	TAGCCAGACC	37 tcattccgcgaagcggcatttcgcctatacacgtcct 1<br>1515 tcattccgcgaagcggcatttcgcctatacacgtcct 1551
sp7	KP233178	*cap*	91.7	$1.3\cdot10^{-11}$	CGATCCTCCA	TAGTCTAAAC	36 caggatggttttagacgtgatgctgtccctttgtat 1<br>772 caagatggttttagacgtgatgctgttccgttgtat 807
sp8	KP264968	-	86.5	$7.3\cdot10^{-8}$	ACGCATCCCA	TTACCAGCCA	36 tgctgctcgtggtactcttgatgc-tgctatatctct 1<br>283 tactgctcgtggtactctcga-gcgtactatatctct 318
sp9	KP233174	*rep*	100	$2.1\cdot10^{-17}$	CCCCGTGCAA	GAGTATAAAG	37 tcgatagcgctccacacgctcttgctctgtttcgtta 1<br>1897 tcgatagcgctccacacgctcttgctctgtttcgtta 1933
sp12	KP233178	*rep*	89.2	$1.3\cdot10^{-10}$	GCCTAGTCCT	CAAGAAGTAT	37 gtagtttctccttagatgctgttatatcgtaccactt 1<br>2411 gtaatttctctttagacgctgttatgtcgtaccactt 2447
sp19	KP264966	*cap*	85.7	$5\cdot10^{-8}$	ATTCTCACCT	AGTACTGGCA	35 ctgcccgtcgatcccactggactcagttatgaggc 1<br>546 ctccccgtggacccgactggactcagttatgaagc 580
sp20	KP233178	*rep*	100	$2.1\cdot10^{-17}$	CGTAAGCCCA	GTGATTATGT	37 cttcctttagaacatagtcaaaatttcttacatgtgt 1<br>2204 cttcctttagaacatagtcaaaatttcttacatgtgt 2240
sp21	KP233178	-	94.4	$2.9\cdot10^{-13}$	ACGAAACCCA	TTACCAGCCA	36 tattgctcgttctactctcgatactgttatatctgc 1<br>277 tactgctcgttctactcttgatactgttatatctgc 312
sp22[e]	KP233175	-	90.5	$1.5\cdot10^{-2}$			1 ctgtcagactgatacggtctg 21<br>1490 ctgactgactgatacggtctg 1510
sp23	KP233184	*rep*	100	$2.1\cdot10^{-17}$	AGACAGTCCA	ACTTATTGTC	37 cgtactttggatactcatttgtaaaaacaaacactct 1<br>1785 cgtactttggatactcatttgtaaaaacaaacactct 1821
sp24	KP233186	*cap*	100	$2.1\cdot10^{-17}$	GTAGACCCCA	TGGTGAGGAT	1 gagaccaatttaacccaggacttgttagaatcaccaa 37<br>604 gagaccaatttaacccaggacttgttagaatcaccaa 640
sp25	KP233181	*rep*	100	$8.6\cdot10^{-17}$	ACGCTATCCG	AGACCTGAAA	36 actaagtgctgttgccagggtcttaactgagcattt 1<br>2126 actaagtgctgttgccagggtcttaactgagcattt 2161
sp26	KP233178	*cap*	77.8	$1.3\cdot10^{-4}$	CTATTATCTA	TTAAGTTGCC	36 atggagttccttcttcggcttttcttcctattgagt 1<br>1304 atggagttccttctggtaccttttttacctgttgagt 1339
sp27	KP233179	*cap*	89.2	$1.3\cdot10^{-10}$	TAGAACTCCT	CTTACAAGAC	1 cccgaagttgatcttgttactgtagttctacctgctg 37<br>1099 cccgaagttgatctgcttactgtagttctccctgccg 1135
sp32[e]	KC545227	*rep*	85.7	$5.9\cdot10^{-3}$			29 agacgagtgcgtgcct-atttggaacaa 3<br>2250 agatgagtgcgtgcctgattt-gatcaa 2276
sp35	KY086298	*rep*	80.6	$3.1\cdot10^{-5}$	TGCACTAACC	CTCCTCTTAT	36 taaaaatattggtaatgtcaaatacatatcctaaag 1<br>677 taaaggtcttggtaatgacaaacacatatcc--aag 710
sp36	KP264968	*cap/-*	75.6	$4.8\cdot10^{-4}$	TGAGACACCG	TGCATGGAGT	1 tgagcgtgtattacaacgg--acg---tgagcttgttgaaa 36<br>1360 tgagcgtgtattacaacggtaaagaaatgatcgagatgaaa 1400
sp41	KP233178	*cap*	88.9	$4.7\cdot10^{-10}$	TGCTGTTCCG	TTCCCCGGCT	1 ttatactggagaattggtcagttacatcaggataat 36<br>802 ttgtattggaggatcggtcagttacatcaggataat 837
sp48	KY086298	*rep*	88.9	$4.7\cdot10^{-10}$	CATAATCCCA	GTTTATCTTG	36 atacctgaagctcatgtagaagaagcaacagactca 1<br>226 attcctgaagctcatgtagaagaagcaatggacaca 261
sp49[e]	KT600068	*cap*	83.9	10^{-3}			28 gattatgtt--gcctg-tattgataccatgg 1<br>655 gcttgtgttatgcctgctattgataccatgg 685
sp56	KY086298	*rep*	82.5	$9.3\cdot10^{-7}$	AGGGACACCA	GAAGGGTTAA	1 aaagaaatcatacaatgggttcatagcag-ttataaaaat 39<br>505 aaagagatcatacaatgggttcat-tcagcatatgataat 543
sp67	KP233178.	*cap*	83.8	$1.1\cdot10^{-7}$	AGGCTCACCA	TTGGGTCAAC	37 gtgacatgttcaaccctggtcttgttcgcataacgaa 1<br>626 gcgacatgttcaatccgggcctagtcagaatcacgaa 662
sp69[e]	KC545226	-	85.2	$1.2\cdot10^{-3}$			36 tagtctttatatatttgtttgttcatt 10<br>2501 ttgtgtttatatatttgtttgattatt 2527
sp72	KY086298	*rep*	80.6	$7.3\cdot10^{-6}$	ATATACACCT	TCTACATGAG	36 agaagcaacagactcttgggaatatgaaaggaaaga 1<br>246 agaagcaatggacacatgggattatgagagaaaaga 281
sp74	KC545226.	*cap*	77.8	$1.3\cdot10^{-4}$	ATTGGTGCCT	TATGCGACAG	1 tcgtctactctgcctgtagatcctactggtttgagt 36<br>663 gcaagtactttgcctgtggatccaacaggtttgagt 698
sp79	KM573770	*rep*	75	$6.2\cdot10^{-3}$	TAACGACACG	ATGGTATCAG	1 cccaatattcgcgcgcgaactgttccgacaggccag 36<br>301 cccaatattctc-cgcgtacggttcggacacatgag 335

[a] Intergenic sequences are indicated by a minus sign (-).

[b,c] Adjacent region in the subject next to a local-global alignment. Upstream and downstream refer to transcription of crRNA. Correspondence with PAM sequence is underlined.

[d] In the alignments the spacer sequence is represented above.

[e] Local alignment. The entire length of the spacer is not included.

Supplementary Table 2. *Methanomassiliicoccales* sequences where CRISPR have been sought.

Accessions	Description
CP002916.1	Thermoplasmatales archaeon BRNA1, complete genome
CP004049.1	Candidatus Methanomethylophilus alvus Mx1201, complete genome
NC_020913.1	Candidatus Methanomethylophilus alvus Mx1201, complete genome
CP005934.1	Candidatus Methanomassiliicoccus intestinalis Issoire-Mx1, complete genome
NC_021353.1	Candidatus Methanomassiliicoccus intestinalis Issoire-Mx1, complete genome
CP010070.1	Candidatus Methanoplasma termitum strain MpT1, complete genome
NZ_CP010070.1	Candidatus Methanoplasma termitum strain MpT1, complete genome
CP013703.1	Methanogenic archaeon mixed culture ISO4-G1, complete genome
CP014214.1	Methanogenic archaeon ISO4-H5 chromosome, complete genome
NZ_CP014214.1	Methanogenic archaeon ISO4-H5 chromosome, complete genome
LJKK00000000.1	Methanomassiliicoccales archaeon RumEn M1, whole genome shotgun sequencing project
LJKK01000001-LJKK01000182	Methanomassiliicoccales archaeon RumEn M1, whole genome shotgun sequence contigs
LJKL01000001-LJKL01000018	Methanomassiliicoccales archaeon RumEn M2, whole genome shotgun sequence contigs
CAJE01000001-CAJE01000026	Methanomassiliicoccus luminyensis B10 WGS whole genome shotgun sequence contigs
NZ_CAJE00000001-NZ_CAJE01000026	Methanomassiliicoccus luminyensis B10, whole genome shotgun sequence contigs
LOPS01000001-LOPS01000050	Candidatus Methanomethylophilus sp. 1R26, whole genome shotgun sequence contigs
NZ_LOPS01000001-NZ_LOPS01000050	Candidatus Methanomethylophilus sp. 1R26, whole genome shotgun sequencing project contigs

Supplementary Table 3. Description of unambiguous CRISPR loci found in *Methanomassiliicoccales* organized by their consensuses.

CRISPR 1	Consesus:"gttagaaatccatctaaactagaatgtaaat"	2 Arrays			113 spacers (112 unique)	
Accession	Description	Array Location	Repeats	Period*	Dev**	Consensus:
CP005934, NC_021353	Candidatus Methanomassiliicoccus intestinalis Issoire-Mx1, complete genome	930280..937791	112	67,4	0,63	gttagaaatccatctaaactagaatgtaaat
		940586..940756	3	68	0	tttcatttacattctagtttagatggatttctaac
CRISPR 2	Consensus:"gtcgcacccttcacgggtgcgtggattgaaac"	3 Arrays			130 spacers (129 unique)	
Accession	Description	Array Location	Repeats	Period*	Dev**	Consensus:
CAJE01000012, NZ_CAJE01000012	Methanomassiliicoccus luminyensis B10 WGS project CAJE00000000 data, contig 00012, whole genome shotgun sequence	732087..739595	114	66,2	0,78	gtcgcacccttcacgggtgcgtggattgaaac
		723955..724114	3	65,3	0,75	tttcaatccacgcacccgtgaagggtgcgac
LOPS01000002, NZ_LOPS01000002	Candidatus Methanomethylophilus sp. 1R26 contig_10, whole genome shotgun sequence	64339..64479	3	66,5	0,5	gtcgcaccccctcgcgggtgcgtggattgaaac
LOPS01000033, NZ_LOPS01000033	Candidatus Methanomethylophilus sp. 1R26 contig_37, whole genome shotgun sequence	7715..8616	14	66,9	0,72	gtttcaatccacgcacccgcgaggggtgcgac
CRISPR 3	Consensus:" ctcaaaactcattcgaatctctactctttgtagat "	1 Array			53 spacers (53 unique)	
Accession	Description	Array Location	Repeats	Period*	Dev**	Consensus:
CP010070, NZ_CP010070	Candidatus Methanoplasma termitum strain MpT1, complete genome	1021325..1024609	54	61,3	0,66	ctcaaaactcattcgaatctctactctttgtagat
CRISPR 4	Consensus:"gagttccccacgcatgtggggatgaaccg"	1 Array			33 spacers (33 unique)	
Accession	Description	Array Location	Repeats	Period*	Dev**	Consensus:
CP014214	Methanogenic archaeon ISO4-H5 chromosome, complete genome	1154017..1156049	34	60,97	0,06	gagttccccacgcatgtggggatgaaccg
CRISPR 5	Consensus:"atctacactagtagaaattctgaatgagttttagac"	1 Array			47spacers (43 unique)	
Accession	Description	Array Location	Repeats	Period*	Dev**	Consensus:
CP004049, NC_020913	Candidatus Methanomethylophilus alvus Mx1201, complete genome	867719..870703	48	62,7	1,04	atctacactagtagaaattctgaatgagttttagac
CRISPR 6	Consensus:"gtttgagagtgttgtcaaatacgaggtaactcaatc"	1 Array			6 spacers (6 unique)	
Accession	Description	Array Location	Repeats	Period*	Dev**	Consensus:
CP002916	Thermoplasmatales archaeon BRNA1, complete genome	675993..676425	7	66,2	0,28	gtttgagagtgttgtcaaatacgaggtaactcaatc
CRISPR 7	Consensus:"cgccttccaaggctgcacctccctgacgtccatcac"	1 Array			5 spacers (5 unique)	
Accession	Description	Array Location	Repeats	Period*	Dev**	Consensus:
LJKK01000086	Methanomassiliicoccales archaeon RumEn M1 contig00032, whole genome shotgun sequence	11338..11718	6	69	0	cgccttccaaggctgcacctccctgacgtccatcac

* Period is the distance (nt) between the same base in consecutive repetitions

** Dev is the absolute deviation of period across the array.

Supplementary Table 4. Sequences of *Smacoviridae* analysed in this work*.

Accession	Description
GQ351272	Chimpanzee stool associated circular ssDNA virus isolate DP152, complete genome
GQ351273	Chimpanzee stool associated circular ssDNA virus isolate GM495, complete genome
GQ351274	Chimpanzee stool associated circular ssDNA virus isolate GM476, complete genome
GQ351275	Chimpanzee stool associated circular ssDNA virus isolate GM510, complete genome
GQ351276	Chimpanzee stool associated circular ssDNA virus isolate GM488, complete genome
GQ351277	Chimpanzee stool associated circular ssDNA virus isolate GM415, complete genome
JN634851	Circoviridae bovine stool/BK/KOR/2011 isolate CP11-49-3, complete genome
JX274036	Porcine stool-associated circular virus isolate Cass, complete genome
KC545226	Porcine stool-associated circular virus 2 isolate f, complete genome
KC545227	Porcine stool-associated circular virus 3 isolate 3L7, complete genome
KC545228	Porcine stool-associated circular virus 3 isolate 4L13, complete genome
KC545229	Porcine stool-associated circular virus 3 isolate 4L5, complete genome
KC545230	Porcine stool-associated circular virus 3 isolate L2T, complete genome
KF193403	PoSCV Kor J481, complete genome
KF880727	Turkey stool associated circular ssDNA virus strain TuSCV, complete genome
KJ577810	Porcine stool-associated circular virus 1 isolate DP2, complete genome
KJ577811	Porcine stool-associated circular virus 1 isolate DP3, complete genome
KJ577812	Porcine stool-associated circular virus 7 isolate EP2-A, complete genome
KJ577813	Porcine stool-associated circular virus 7 isolate EP2-B, complete genome
KJ577814	Porcine stool-associated circular virus 7 isolate EP3-C, complete genome
KJ577815	Porcine stool-associated circular virus 7 isolate EP3-D, complete genome
KJ577816	Porcine stool-associated circular virus 9 isolate FP1, complete genome
KJ577817	Porcine stool-associated circular virus 8 isolate GP2, complete genome
KJ577818	Porcine stool-associated circular virus 2 isolate TP3, complete genome
KJ577819	Porcine stool-associated circular virus 6 isolate XP1, complete genome
KM573769	Dromedary stool-associated circular ssDNA virus isolate DcSCV_c1359, complete genome
KM573770	Dromedary stool-associated circular ssDNA virus isolate DcSCV_c1072, complete genome
KM573771	Dromedary stool-associated circular ssDNA virus isolate DcSCV_c1345, complete genome
KM573772	Dromedary stool-associated circular ssDNA virus isolate DcSCV_c1378, complete genome
KM573774	Dromedary stool-associated circular ssDNA virus isolate DcSCV_c1330, complete genome
KM573775	Dromedary stool-associated circular ssDNA virus isolate DcSCV_c1358, complete genome
KM598409	Odonata-associated circular virus-21 isolate OdasCV-21-US-1679SC3-12, complete genome
KM598410	Odonata-associated circular virus-5 isolate OdasCV-5-US-1683LM1-12, complete genome
KP233174	Human smacovirus 1 isolate France/8/2008/2444, complete genome
KP233175	Human smacovirus 1 isolate France/1/2008/2610, complete genome
KP233176	Human smacovirus 1 isolate France/8/2008/2449, complete genome
KP233177	Human smacovirus 1 isolate France/4/2009/4265, complete genome
KP233178	Human smacovirus 1 isolate France/1/2009/3664, complete genome
KP233179	Human smacovirus 1 isolate France/1/2009/3663, complete genome
KP233180	Human smacovirus 1 isolate Oregon/6/2011/GottageGrove/5A1, complete genome
KP233181	Human smacovirus 1 isolate Oregon/6/2011/GottageGrove/B3, complete genome
KP233182	Human smacovirus 1 isolate Oregon/6/2011/GottageGrove/B45, complete genome
KP233183	Human smacovirus 1 isolate Oregon/8/2011/Portland/D56, complete genome
KP233184	Human smacovirus 1 isolate Virginia/1/2012/Mecklenburg/H19, complete genome
KP233185	Human smacovirus 1 isolate Virginia/2/2012/Albemarle/5I17, complete genome
KP233186	Human smacovirus 1 isolate Virginia/2/2012/Chesapeake/J23, complete genome
KP233187	Human smacovirus 1 isolate Virginia/2/2012/Middlesex/I22, complete genome
KP233188	Human smacovirus 1 isolate Virginia/12/2011/Albemarle/G16, complete genome
KP233189	Black howler monkey smacovirus isolate SF1, complete genome
KP233190	Chimpanzee smacovirus isolate SF2, complete genome

KP233191	Gorilla smacovirus isolate SF3, complete genome
KP233192	Gorilla smacovirus isolate SF4, complete genome
KP233193	Human smacovirus 1 isolate Orgeon/8/2011/Portland/D53, complete genome
KP233194	Lemur smacovirus isolate SF5, complete genome
KP264964	Human smacovirus 1 isolate France/2/2008/2548, complete genome
KP264965	Human smacovirus 1 isolate France/3/2008/2623, complete genome
KP264966	Human smacovirus 1 isolate France/12/2008/3454, complete genome
KP264967	Human smacovirus 1 isolate France/12/2008/3454, complete genome
KP264968	Human smacovirus 1 isolate France/3/2009/4191, complete genome
KP264969	Human smacovirus 1 isolate France/6/2008/2871, complete genome
KP860906	Rat stool-associated circular ssDNA virus isolate KS/11/0577, complete genome
KP860907	Rat stool-associated circular ssDNA virus isolate Mu/10/1799, complete genome
KP860908	Rat stool-associated circular ssDNA virus isolate KS/11/0582, complete genome
KT600068	Human feces smacovirus 2 strain SmaCV2, complete genome
KT600069	Human feces smacovirus 3 strain SmaCV3, complete genome
KT862218	Bovine faeces associated smacovirus 2 isolate 23_Fec30587_cow, complete sequence
KT862219	Sheep faeces associated smacovirus 3 isolate 47_Fec58091_sheep, complete sequence
KT862220	Sheep faeces associated smacovirus 1 isolate 47_Fec58729_sheep, complete sequence
KT862221	Sheep faeces associated smacovirus 2 isolate 47_Fec60415_sheep, complete sequence
KT862222	Bovine faeces associated smacovirus 3 isolate 48_Fec5_cow, complete sequence
KT862223	Bovine faeces associated smacovirus 1 isolate 48_Fec59973_cow, complete sequence
KT862224	Bovine faeces associated smacovirus 5 isolate 48_Fec9_cow, complete sequence
KT862225	Porcine faeces associated smacovirus 1 isolate 49_Fec25_pig, complete sequence
KT862226	Porcine stool-associated circular virus isolate 56_Coc3310_hare, complete sequence
KT862227	Porcine stool-associated circular virus isolate 59_Coc3310_possum, complete sequence
KT862228	Bovine faeces associated smacovirus 4 isolate GP3_45917_cow, complete sequence
KT862229	Bovine faeces associated smacovirus 6 isolate GP3_46075_cow, complete sequence
KX838317	Human feces-associated smacovirus isolate BWA1115, complete genome
KX838318	Human feces-associated smacovirus isolate BWA7684, complete genome
KY086298	Chicken associated smacovirus strain RS/BR/2015/1, complete genome
KY086299	Chicken associated smacovirus strain RS/BR/2015/4, complete genome
KY086300	Chicken associated smacovirus strain RS/BR/2015/3, complete genome
KY086301	Chicken associated smacovirus strain RS/BR/2015/2, complete genome

* Dataset selected from a previous paper[1].

Supplementary reference

1. Varsani, A. & Krupovic, M. Smacoviridae: a new family of animal-associated single-stranded DNA viruses. *Arch. Virol.* **163,** 2005–2015 (2018).
